# Animals and their products utilized as medicines by the inhabitants surrounding the Ranthambhore National Park, India

**DOI:** 10.1186/1746-4269-2-46

**Published:** 2006-11-03

**Authors:** Madan Mohan Mahawar, DP Jaroli

**Affiliations:** 1 Department of Zoology, Government Post Graduate College, Sawai Madhopur, Rajasthan, India; 2 Department of Zoology, University of Rajasthan, Jaipur, Rajasthan, India

## Abstract

The present ethnozoological study describes the traditional knowledge related to the use of different animals and animal-derived products as medicines by the inhabitants of villages surrounding the Ranthambhore National Park of India (Bawaria, Mogya, Meena), which is well known for its very rich biodiversity. The field survey was conducted from May to July 2005 by performing interviews through structured questionnaires with 24 informants (16 men and 8 women), who provided information regarding therapeutic uses of animals. A total of 15 animals and animal products were recorded and they are used for different ethnomedical purposes, including tuberculosis, asthma, paralysis, jaundice, earache, constipation, weakness, snake poisoning. The zootherapeutic knowledge was mostly based on domestic animals, but some protected species like the collared dove (*Streptopelia sp*.), hard shelled turtle (*Kachuga tentoria*), sambhar (*Cervus unicolor*) were also mentioned as important medicinal resources. We would suggest that this kind of neglected traditional knowledge should be included into the strategies of conservation and management of faunistic resources in the investigated area.

## Background

The healing of human ailments by using therapeutics based on medicines obtained from animals or ultimately derived from them is known as zootherapy [1]. As Marques states, "all human culture which presents a structured medical system will utilize animals as medicines" [2]. The use of animals for medicinal purposes is part of a body of traditional knowledge which is increasingly becoming more relevant to discussions on conservation biology, public health policies, sustainable management of natural resources, biological prospection, and patents [3]. Research interest and activities in the areas of ethnobiology and ethnomedicine have increased tremendously in the last decade. Since the inception of the disciplines, scientific research in ethnobiology and ethnomedicine has made important contributions to understanding traditional subsistence and medical knowledge and practice [4]. But in India the traditional knowledge system is fast eroding due to urbanization. So there is an urgent need to inventorise and record all ethnobiological information among the different ethnic communities before the traditional cultures are completely lost [5]. A lot of work has been done in the Ranthambhore National Park on the medicinal plants & plant products and documented too, but there is a definite scarcity of such knowledge when it comes to animal products. Thus there is an urgent need to make such study in the field of zootherapy and document it, so that it can be put to the welfare of human kind. Therefore keeping this aspect in view, we have undertaken this study.

### Study area

**Figure 1:**
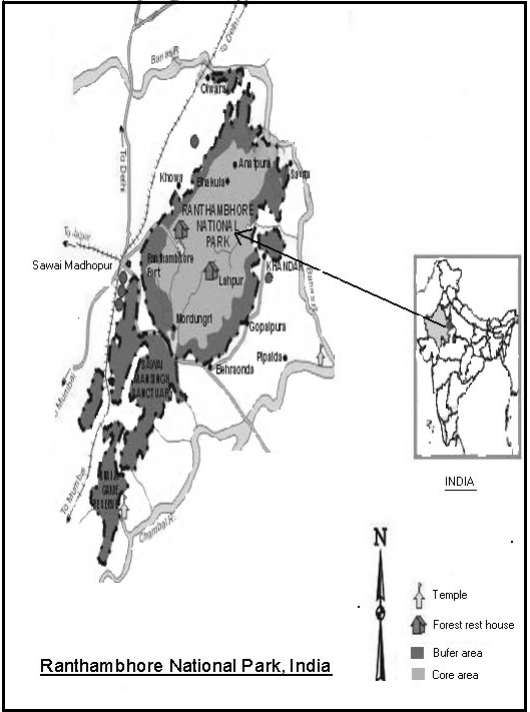
Map of the study areaxsxs.

The Ranthambhore National Park (250 54' N – 260 12' N and 760 22' E – 760 39' E) at the junction of the *Aravalis *and the *Vindhyas *(mountain ranges) is a unique juxtaposition of natural richness, standing out conspicuously in the vast, arid and denuded tract of eastern Rajasthan, barely 14 kilometer from the town, *Sawai Madhopur*. Being a typical representative of dry-deciduous *dhok *(*Anogeissus pendula*) forest, the vegetation of Ranthambhore is considered as i) *Tropical Dry Deciduous Forest *and ii) *Tropical Thorn Forest *[6]. The climate is dry-subtropical with four distinct seasons: summer (March to June), followed by monsoon (July-August), post-monsoon (September-October) and winter (November-February). Average annual rainfall of about 800 mm is received mostly during July-September. From socio-cultural point of view, the region exhibits a great ethnic and cultural diversity. The present study was mainly conducted in the villages surrounding Ranthambhore national park, Rajasthan (figure 1). Most of the information was collected from *Mogya *and *Bawaria *people, which are living around the national park.

## Methodology

Data were obtained through field survey conducted from May to July 2005 by performing interview through structured questionnaire with 24 selected people (*Informants*), to collect information about traditional knowledge regarding use of animals and their products. These informants were local herbalists, healers, farmers, and midwives. The Informants are between 40–74 age groups. The selection of *Informants *was based on their recognition as experts and knowledgeable members concerning folk medicine. We ask the informants whether they use animals in the healing practices. Then we ask that which animal remedies have been prescribes for which ailment. We also ask the modes of preparation of remedies and how the medicines are administered, since this kind of information indicates how a given medicine can be therapeutically efficient in terms of the right ingredients, the proper dose, and the right length of preparation. According to them, their knowledge of folk medicine was acquired mainly through parental heritage, or because they have experience about medicinal value of animals to heal their kin or themselves. The interviews were recorded and documented. All the animal species were identified by using relevant and standard literature.

## Result and discussion

The present study describes the traditional knowledge of treating various kinds of disease using different animals and their product by inhabitants (*Bawaria, Mogya*, *Meena *etc) of villages surrounding the Ranthambhore National Park of India. The information of all local names of the animal, part or product used to cure and methods of preparation were provided by the *Informants*. In this study, we enlisted 15 animal species, which are being used for 20 medicinal purposes. These animals are used as whole or body part or byproduct like milk, blood, organ, skeleton etc. for the treatment of different kind of ailments including tuberculosis, asthma, paralysis, jaundice, earache, constipation, weakness, snake poison etc. (see table 1).

**Table 1:** List of animals and their parts use for therapeutic purpose in the studied area.

English Name	Scientific Name	Local Name	Part used	No. of Informants reporting the use	Method of preparation and medicinal use	Related earlier reported use in India [Ref.]
1. Indian ass	*Equs hemionus*	Gadha	Dung	2	Dung kept in water and after one day filtered water is given to cure jaundice.	
2. Cow	*Bos indicus*	Gai	Urine	18	Weakness due to fever is cure by drinking urine.	
			Urine	2	Given to cure cancer.	
			Dung + Milk	20	Muscle pain can relieve by smear of dung and milk mixture.	The Dried dung is burnt and ash is applied to treat utricaria in Kachchh [15].
			Ghee	5	250 gm Ghee + 100 gm Black pepper mixture given orally to neutralize snake poison.	
3. Dog	*Canis familiaris*	Kukaro	Urine	22	Used as eardrop for curing earache.	Also reported by Naga tribe of Nagaland [11].
4. Goat	*Capra indicus*	Bakri	Urine	3	Urine of goat administered orally to cure tuberculosis.	Reported by Ao [12] and Naga [11] tribe for asthma, T.B., paralysis, and by Tamilnadu tribe for insect bite [13].
			Milk	22	Mouth ulcer is treated by direct spray of milk from breast of goat to tongue of a patient.	
5. Human	*Homo sapiens*	Manakh	Urine	24	Human urine is used as antiseptic for wound healing.	Also reported by Naga tribe of Nagaland [11].
6. Indian Peafowl	*Pavo cristatus*	Mor	Leg	5	Peacock's leg is rubbed with water and this essenced water is used in ear infections	Also reported by Naga tribe of Nagaland [11], Bhil of Rajasthan [19]. Legs boil with oil in kachchh [15] and Maharastra [17] for similar purpose.
7. Pig	*Sus scrofa*	Soor	Fat	12	Fat of pig is use as massage cream in muscular pain.	Also reported by Ao tribe of Nagaland [12], but fat of pig used for Hemorrhoids in Tamilnadu [13].
8. Sambhar	*Cervus unicolor*	Sambhar	Antler	2	Antler is rubbed with water this paste is applied in eye ailments.	Also reported in Kachchh of Gujrat [15].
9. Sheep	*Capra sp*.	Menda	Milk	23	Used as massage cream in muscular pain.	
10. House sparrow	*Passer domesticus*	Cheedi	Fecal	20	Fecal matter is applied in the anus of baby to treat constipation.	Ash of excreta is used for treatment of asthma in children is reported in Kchchh [15].
11. Pigeon	*Columba livia*	Kabutar	Fresh blood	14	The fresh blood is massaged externally to treat paralysis.	Same use reported in Kachchh [15] and Tamilnadu [13].
12. Collared dove	*Streptopelia sp*.	Kamedii	Flesh	3	To attain early puberty girls eat flesh of collared dove.	
13. Hardshelled Turtle.	*Kachuga tentoria*	Kachhua	Carapace	7	Ash of carapace is used in lung diseases as cough, asthma, T. B. etc.	Ash of *Lissemys punctatus*' Carapace is used for healing of internal injuries, pruritis and cough (Kachchh) [15].
14. Honey bee	*Apis indica*	Mokh	Honey	21	Used as eye drops to cure eye disease.	Honey is used for cough and could. (Tamilnadu tribes) [13] [16].
15. Bivalves	*Mactra sp*.	Seepi	Shell	3	Shell of sepia is rubbed with clarified butter (*ghee*) and red lead (*sindoor*) to apply on acne to cure.	

Since ancient times animals, their parts, and their products have constituted part of the inventory of medicinal substances used in various cultures. This phenomenon is marked by both a broad geographical distribution and very deep historical origins [7]. In Pakistan, 31 substances were listed (animal parts and products), constituting 9% of all the medicinal substances in the inventory of traditional medicines [8]. Costa-Neto describes the use of 180 animal species as medicinal resources in the state of Bahia, Northeastern Brazil [9]. A survey of traditional materia medica in use in the markets of Israel recorded 20 substances of animal origin [25]. In the states of Maranhão and Paraíba (Northeast Brazil) a survey carried out and recorded 100 animal species was used as medicine [26]. Examination and research show that these substances are similar to those used as remedies throughout human history, irrespective of geographical borders [7].

In India, nearly 15–20 percent of the Ayurvedic medicine is based on animal-derived substances. There are references to nearly 380 types of animal substances in *Charaka Samhita *[10]. The Hindu religion has used five products (milk, urine, dung, curd and ghee) of the cow for purification since ancient times [27]. Besides immense knowledge has come down to modern times through folklore as various practices became a part of tradition amongst various groups in India (see table 2). Different animals used by the Naga tribe of Nagaland [11], Ao tribe of Nagaland[12], Irular, Kurimba of Tamilnadu[13], Chakhesang tribe of Nagaland[14], Kachch (Gujrat)[15], Kanikar, Paliyar of Taminadu[16], Bhil, Gamit, Kokna etc of Maharastra[17], Assam[18], Bhil of Rajasthan[19], Dibrugarh (Assam) [20]etc. has some or the other relevance with the animals that are found to be use by the Mogya, Bawaria, Meena etc. residing in this part of India.

**Table 2:** Ethnomedicinal uses of animals reported from different parts of India.

Tribes/Ethnic Groups/Region/Indigenous people	Number of animals Reported	Authors	**Ref. No**.
Assam	5	Dutta A (1996)	[18]
Sporadic study in India	20	Gosh A K, Maiti P K (1996)	[24]
Chakhesang of Nagaland	23	Kakati and Doulo (2000)	[14]
*Bhil *of Rajasthan	17	Sharma S K (2002)	[19]
*Bhil, Gamit, Kokna *etc of Maharastra	15	Patil S H (2003)	[17]
Chhattisgarh	10	Oudhia P (2003)	[23]
Chhattisgarh	7	Oudhia P (2003)	[21]
Bhopalpatnam (chhattisgarh)	3	Oudhia P (2003)	[22]
Kachch (Gujrat)	34	Gupta Leena et al (2003)	[15]
*Irular, Kurimba *of Tamilnadu	26	Solvan A et al (2004)	[13]
*Kanikar, Paliyar *of Taminadu	11	Ranjit Singh ASA (2004)	[16]
*Naga *tribe of Nagaland	26	Jamir N S et al (2005)	[11]
Dibrugarh (Assam)	4	Dilip Kalita (2005)	[20]
*Ao *tribe of Nagaland	25	Kakati L N et al (2006)	12]
*Mogya, Meena, Bawaria *of Rajasthan	15	Mahawar, Jaroli (Present study)	

The use of urine drop of *Canis familiaris *against earache has been also reported amongst the Naga tribe of Nagaland [11]. The urine of *Capra indicus *has been also reported by Ao and Naga tribes for asthma, T.B., paralysis, [11,12] but the milk of this animal is use for mouth ulcer has never been reported earlier. *Pavo cristatus' *legs uses for ear infection are also similar in Naga tribe of Nagaland and Bhil of Rajasthan, [11,19] but Legs are boil with oil in kachchh and Maharastra for similar purpose [15,17]. Ao tribe of Nagaland [12] also reports the fat of *Sus scrofa *in muscular pain, but in Tamilnadu this is used for Hemorrhoids [13]. The use of antler of *cervus unicolor *for eye ailments and the fresh blood of *columba livia *for paralysis has been also reported in the Kachchh region of Gujarat [15]. The use of fecal matter of *Passer domesticus *to treat baby constipation, but ash of excreta is used for treatment of asthma in children is reported in Kachchh[15]. The flesh of *Streptopelia sp*. to attain early puberty and dung of *Equs hemionus *to cure jaundice has never been reported earlier in India. Ash of *Kachuga tentoria*' carapace is used in lung diseases as cough, asthma, T. B. etc. but *Lissemys punctatus*' Carapace is used for healing of internal injuries, prurities and cough in Kachchh region [15].

**Figure 2:**
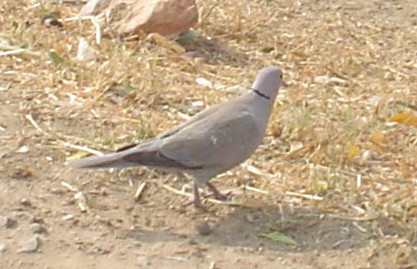
Collared dove.

Noteworthy is the observation that mostly animal byproducts are used in traditional health care systems without any loss to animal. The therapeutic information's is mostly based on domestic animals, but some protected species like the collared dove (*Streptopelia sp*) (figure 2), hard shelled turtle (*Kachuga tentoria*), sambhar (*Cervus unicolor*) are also included as important medicinal resources in this studied area. Kakati and Doulo enlisted six species as rare among twenty three species among Chakhesang tribe of Nagaland[14]. Inadequate knowledge and myths associated with the therapeutics like children are made to wear beer's claw around their neck as it is suppose to protect them from evil forces, similarly animals like sheep and goat are sacrifice to cause healing as this act is believe to please local gods. However such acts have cause harm to animal life. Thus there is a need to shift the focus from how to obtain the greatest amount of zootherapeutical resources to how to ensure future uses. There is also a need for a transdisciplinary approach to integrate the various aspects of zootherapy in such a way that frameworks or methods to amalgamate ecological and social components of that practice can be increasingly tested [3]. So the traditional knowledge should be included into the strategies of conservation and management of faunistic resources [26]. Further studies are required not only to confirm the presence of bioactive compounds in these traditional remedies, but also to emphasize more sustainable use of these resources.
